# Chemical dissection of the cell cycle: probes for cell biology and anti-cancer drug development

**DOI:** 10.1038/cddis.2014.420

**Published:** 2014-10-16

**Authors:** S Senese, Y C Lo, D Huang, T A Zangle, A A Gholkar, L Robert, B Homet, A Ribas, M K Summers, M A Teitell, R Damoiseaux, J Z Torres

**Affiliations:** 1 Department of Chemistry and Biochemistry, University of California, Los Angeles, CA, USA; 2 Department of Bioengineering, University of California, Los Angeles, CA, USA; 3 Department of Medicine (Division of Hematology-Oncology), David Geffen School of Medicine, University of California, Los Angeles, CA, USA; 4 Department of Molecular and Medical Pharmacology, David Geffen School of Medicine, University of California, Los Angeles, CA, USA; 5 Department of Surgery (Division of Surgical-Oncology), David Geffen School of Medicine, University of California, Los Angeles, CA, USA; 6 Jonsson Comprehensive Cancer Center, University of California, Los Angeles, CA, USA; 7 The Department of Cancer Biology, Lerner Research Institute, Cleveland, OH, USA; 8 Department of Pathology and Laboratory Medicine, David Geffen School of Medicine at the University of California, Los Angeles, CA, USA; 9 Broad Stem Cell Research Center, University of California, Los Angeles, CA, USA; 10 California NanoSystems Institute, University of California, Los Angeles, CA, USA; 11 Molecular Biology Institute, University of California, Los Angeles, CA, USA

## Abstract

Cancer cell proliferation relies on the ability of cancer cells to grow, transition through the cell cycle, and divide. To identify novel chemical probes for dissecting the mechanisms governing cell cycle progression and cell division, and for developing new anti-cancer therapeutics, we developed and performed a novel cancer cell-based high-throughput chemical screen for cell cycle modulators. This approach identified novel G1, S, G2, and M-phase specific inhibitors with drug-like properties and diverse chemotypes likely targeting a broad array of processes. We further characterized the M-phase inhibitors and highlight the most potent M-phase inhibitor MI-181, which targets tubulin, inhibits tubulin polymerization, activates the spindle assembly checkpoint, arrests cells in mitosis, and triggers a fast apoptotic cell death. Importantly, MI-181 has broad anti-cancer activity, especially against BRAF^*V600E*^ melanomas.

The cell cycle is a set of coordinated events that culminate in the formation of two cells from one mother cell. It's composed of four major phases; G1 (growth phase 1), S (DNA synthesis phase), G2 (growth phase 2) and M (mitosis), which function to integrate environment sensing signaling pathways with cell growth and proliferation.^1^ Cancer cells often deregulate the cell cycle and undergo unscheduled cell divisions, therefore inhibition of the cell cycle represents an opportunity for therapeutic intervention in treating proliferative diseases like cancer.^2^ Most anti-cancer drugs perturb the proliferation cycle of tumor cells by inhibiting/damaging cell cycle events, which activate checkpoints, arrest cells and induce apoptosis.^3^ For example, inhibitors targeting DNA replication (5-fluorouracil) and cell division (microtubule-stabilizing paclitaxel) have been used successfully for treating a broad array of cancers including breast and colorectal cancers.^2^ Nevertheless, due to toxicity issues, drugs targeting the cell division machinery like mitotic kinases (AurKA/B and Plk1) and kinesins (Kif11 and CENP-E) have been developed.^3^ However, these drugs have shown limited efficacy *in vivo.*^4^ Thus, there is a critical need to identify novel drug-like molecules that inhibit cancer cell cycle progression, which can be developed into novel cancer therapies.

Genome wide studies aimed at depleting the expression of human genes and characterizing their contribution to cell cycle progression have generated a wealth of information regarding the enzymatic machinery required for proliferation.^5^ These enzymes have become the focus of targeted screening campaigns aimed at finding inhibitors to their activities. For example, an *in vitro* chemical screen targeting Plk1 identified the small molecule BI2536.^6^ BI2536 was not only used to define novel roles for Plk1 during cell division, it was further developed into an anti-cancer drug whose efficacy is being evaluated in clinical trials.^7^ Therefore, beyond their therapeutic potential, inhibitors can be used as molecular probes for dissecting the function of enzymes critical for cell cycle progression in an acute and temporal manner. However, there are no inhibitors to the majority of the cell cycle machinery and the discovery and characterization of such inhibitors would aid our ability to understand the mechanisms regulating cell division.

Although molecularly targeted screens have grown in popularity, they rely on the previous identification and validation of specific cancer targets with druggable activities/interactions.^8^ As an alternative, unbiased high-throughput chemical screens have tried to identify inhibitors to a single cell cycle phase,^9, 10, 11, 12, 13, 14, 15^ which limited their ability to identify novel anti-proliferative agents to other phases of the cell cycle. Nonetheless, G2-phase, M-phase, and cytokinesis screens successfully identified inhibitors to Kif11, Plk1, RhoA, and microtubules.^9, 10, 11, 12, 13, 14, 15^ These inhibitors aided the functional characterization of these proteins and were instrumental for developing drugs with therapeutic potential. However, these screens were conducted with a limited number of compounds (100–38 000) or cell extract fractions, with several screens using the same library of 16 320 compounds, thus limiting compound diversity, chemical coverage, and opportunities for novel discoveries. Most screens also lacked chemical analyses to understand the physiochemical properties of bioactive compounds and their cellular targets. In addition, previous screens have not analyzed the four phases of the cell cycle as a biological system. Thus, there is a critical need to develop new screening strategies to discover novel anti-cancer drugs.

This, prompted us to establish an integrated high-throughput screening cell-based strategy for identifying small molecule cell cycle modulators, for use in dissecting the mechanisms of cancer cell division, and for developing novel cancer therapies. We report the development of this novel cell-based screening platform, the discovery of cell cycle phase specific inhibitors, the chemical analyses of these inhibitors, the cell culture characterization of cell division inhibitors, and the detailed examination of MI-181, which has potent anti-cancer activity, especially against melanomas.

## Results

### Discovery of cell cycle modulators

**Figure 1:**
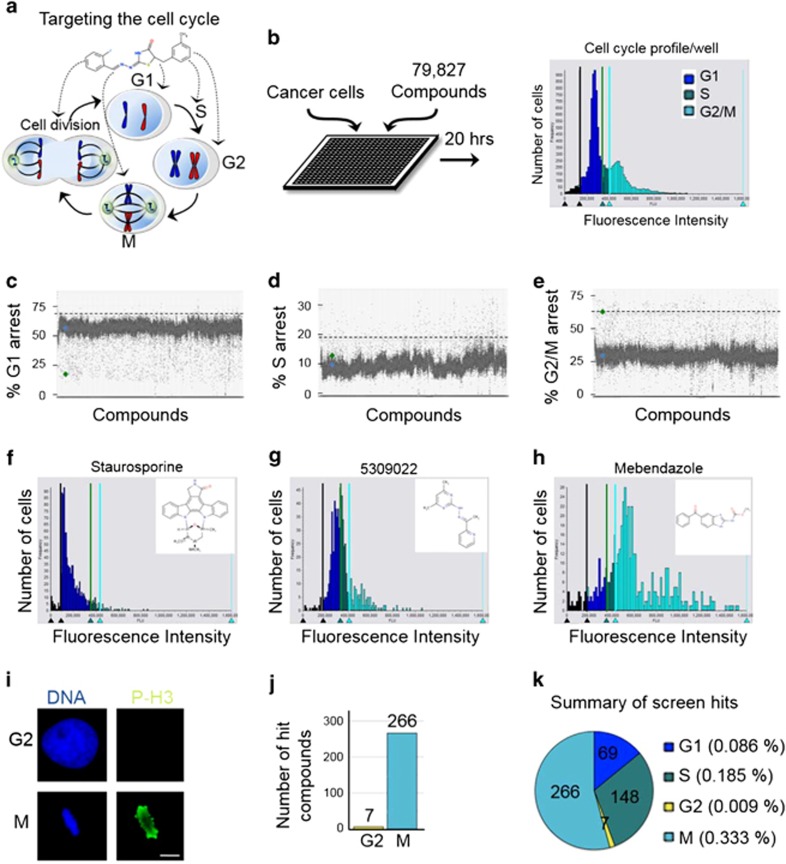
Identification of cell cycle phase specific inhibitors through a novel cell-based high-throughput small molecule screening approach. (**a**) Targeting the cell cycle in the treatment of cancer. (**b**) Cell-based high-throughput screening (HTS) of 79 827 drug-like molecules for cell cycle modulators. Twenty hours post compound treatment, HeLa cancer cells were fixed and stained with Vybrant DyeCycle green, and a high-content cytometer was used to generate a cell cycle profile for each compound. (**c**–**e**) Scatter plots of percent G1, S, and G2/M arrest for all compounds. The cutoffs for G1-phase and S-phase inhibitors were set at >4 and >5 S.D. from the mean, respectively. The cutoff for G2/M inhibitors was set at ≥ 67% G2/M arrest. (**f**–**h**) Examples of G1, S, and G2/M-phase arresting compounds and their cell cycle profiles. (**i**) Immunofluorescence HTS assay for distinguishing G2-phase inhibitors from M-phase inhibitors. Cells were co-stained with 1 *μ*g/ml Hoechst 33342 (blue) and Alexa-488-phospho-histone-H3 (p-H3, green). Mitotic cells are positive for p-H3. Bar indicates 5 *μ*m. (**j**) Summary of screen results indicating that 266 compounds arrest cells in mitosis and 7 compounds arrest cells in G2-phase. (**k**) Screen summary plot of percent hit rate indicates that M-phase inhibitors were the most abundant. (**b**–**k**) See also Supplementary Table 2

To discover novel cell cycle phase specific inhibitors, human HeLa cancer cells were plated into 384-well plates and a diverse compound library (79 827 small molecules) encompassing broad chemical space was used to place one compound per well at 10 *μ*M final concentration (Figures 1a and b and Supplementary Table 1). These compounds were pre-selected based on their drug-like properties: predominantly conform to Lipinski's rule of five for acceptable molecular properties for orally active drugs in humans.^16^ Twenty hours later, the cells were fixed and stained with the DNA-selective stain Vybrant DyeCycle Green, which is cell membrane permeant and after binding to DNA emits a fluorescent signal that is proportional to DNA mass when exited at 488 nm.^17^ Plates were scanned with a fluorescence micro-plate cytometer and a cell cycle histogram profile was generated for each well, which had been treated with one compound (Figure 1b). Cell cycle profiles were grouped and ranked according to the extent of G1, S, and G2/M arrest. The top hits from each phase were cherry picked and retested in triplicate and only those testing positive were considered further. In total we uncovered 69 G1-phase inhibitors (>4 S.D. from the mean), 148 S-phase inhibitors (> 5 S.D. from the mean), and 273 G2/M-phase inhibitors (% G2/M ≥ 67%) (Figures 1c and e and Supplementary Table 2). A representative example of each compound class and their associated cell cycle profiles are indicated in Figures 1f–h.

To distinguish between compounds that arrest cells in G2-phase or M-phase, the 273 G2/M compounds were subjected to a secondary high-throughput screen, where cells were fixed and co-stained with the DNA dye Hoechst 33342 and a Alexa-488 fluorescently labeled antibody that recognizes phospho-histone H3 (p-H3), which is specifically phosphorylated in mitosis^18,19^ (Figure 1i). This analysis revealed that 266 compounds arrested cells in mitosis and 7 arrested cells in G2-phase (Figure 1j and Supplementary Table 2). In total, the screen resulted in a 0.613% cell cycle modulator hit rate, with 0.086% G1-phase inhibitors, 0.185% S-phase inhibitors, 0.009% G2-phase inhibitors, and 0.333% M-phase inhibitors (Figure 1k). These results indicated that our cell cycle profile based approach successfully identified inhibitors to all phases of the cell cycle and likely a broad array of targets.

### Compound chemical analysis and target prediction

**Figure 2:**
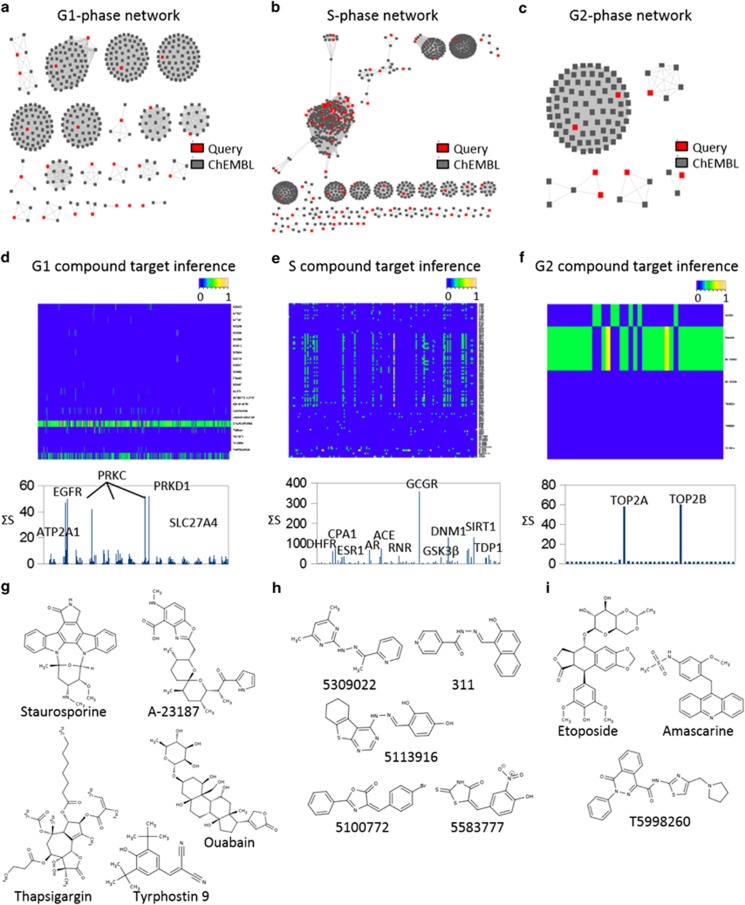
Deconvolving cell cycle modulators. (**a**–**c**) CSNAP network similarity graphs showing 64 G1 chemotypes, 68 S chemotypes, and 5 G2 chemotypes, respectively. Query compounds are in red and ChEMBL compounds are in gray. (**d**–**f**) CSNAP S-score function analyses and prediction of compound on/off-targets. Heatmap summaries of S-scores are scaled from 0–1. The cumulative S-score (∑S-Score) of each assigned target in the target spectrum and the major predicted targets/off-targets are indicated. (**g**–**i**) Highlight of representative compounds within the top clusters for each cell cycle phase. (**a**–**i**) See also Supplementary Table 3

To understand the physiochemical properties of compounds within each cell cycle class, we analyzed the chemical structures of the top compounds from each phase using our newly developed Chemical Similarity Network Analysis Pulldown (CSNAP) computational program. CSNAP searched the ChEMBL database for compounds sharing chemical similarity to hit compounds, retrieved the bioactivity information of each compound found in the ChEMBL database and organized these compounds into network similarity graphs sharing common chemotypes. An implemented scoring function (S-score) was used to score target assignments by counting the target annotation frequency in the nearest neighborhood of query compounds. The predicted compound on/off-targets represented by the S-score were visualized on heatmaps (scaled from 0 to 1) and the most prominent targets were selected from the top peaks, which correlated with the cumulative S-score (∑S-Score) of each assigned target in the target spectrum. This approach previously allowed us to successfully identify major drug targets for M-phase compounds, which included tubulin targeting compounds as well as novel ligands not previously annotated in bioactivity databases. CSNAP analysis of the 69 G1-inhibitors, 148 S-inhibitors, and 7 G2-inhibitors, resulted in the identification of 64 G1 chemotypes, 68 S chemotypes, and 5 G2 chemotypes, respectively. These results indicated that our screening had discovered phase specific and structurally diverse cell cycle inhibitors (Figures 2a–c and Supplementary Table 3).

CSNAP analysis of G1-phase compounds identified kinase inhibitors like Staurosporine, Tyrphostin, and their analogs that mimicked the ATP substrate of PKC and EGFR, which were known to block the MAPK signaling pathway critical for tumor proliferation^20, 21, 22, 23, 24, 25^ (Figures 2d and g). In addition, compounds capable of modulating the intracellular calcium concentration including the ion channel inhibitors Thapsigargin (sarco-endoplasmic reticulum Ca^2+^ ATPase inhibitor), Ouabain (Na^+^/K^+^ ATPase inhibitor), and the ionophore antibiotic A-23187 were also identified^26, 27, 28^ (Figures 2d and g). This was consistent with reports indicating that calcium is an important secondary messenger and that oscillatory calcium signaling is required for MAPK activity and cyclin D1/E synthesis at the G1/S transition.^26^ CSNAP analysis of S-phase compounds indicated that a group of compounds including 5309022 and 5113916 were likely inhibiting ribonucleotide reductase (catalyzes the reduction of ribonucleotides to deoxyribonucleotides; the building blocks for DNA replication and repair^29^) activities by iron chelation through a hydrazone motif similar to that of Triapine and its analog 311^29^ (Figures 2e and h). In addition, two GSK3*β* inhibitors (5100772 and 5583777) were identified among the S-phase inhibitors; consistent with its role in regulating cyclin D1 expression required for S-phase entry and progression^30, 31, 32^ (Figures 2e and h). CSNAP analysis of the seven G2-phase compounds identified DNA topoisomerase II (TOP2) inhibitors including Etoposide and Amsacrine-like analogs^33^ (Figures 2f and i). These DNA intercalating agents trap TOP2 : DNA covalent complexes, which induce DNA damage and G2 checkpoint arrest.^34^ Thus, CSNAP highlighted compounds with known anti-proliferative properties and analogs of these compounds that may be more efficacious. Most importantly, CSNAP identified many novel compounds not sharing chemotype similarity to compounds in bioactivity databases that represent new anti-proliferative agents. Finally, it provided key information for selecting lead compounds with diverse mechanisms of action and targets that perturb distinct cellular pathways essential for cell cycle progression.

### Characterization of M-phase inhibitor potency

**Figure 3:**
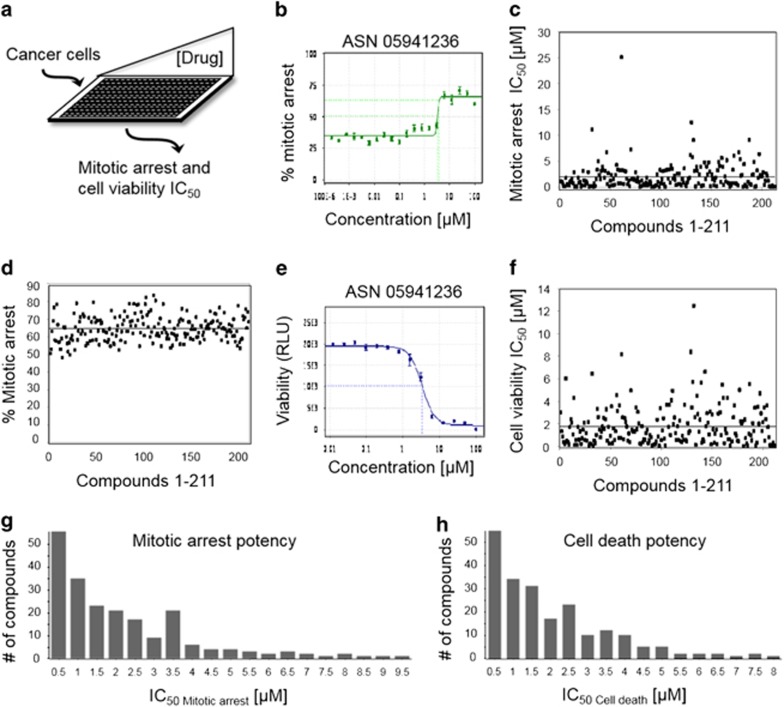
Anti-mitotic compound potency. (**a**) Determination of compound mitotic arrest and cell viability IC_50_. HeLa cells were treated with increasing concentrations of each compound for 20 or 72 h and assayed for mitotic arrest and cell viability, respectively. (**b**) Example of mitotic arrest IC_50_ curve. *x*-axis is drug concentration in *μ*M scale and *y*-axis is percent cells arrested in mitosis. (**c**) Scatter plot of mitotic arrest IC_50_ in *μ*M scale (*y*-axis) for all mitotic compounds (*x*-axis). (**d**) Scatter plot of the percent mitotic arrest IC_50_ in *μ*M scale (*y*-axis) for all mitotic compounds (*x*-axis). (**e**) Example of the cell viability IC_50_ curve. *x*-axis is drug concentration in *μ*M scale and *y*-axis represents cell viability in relative light units (RLU). (**f**) Scatter plot of the cell viability IC_50_ in *μ*M scale (*y*-axis) for all mitotic compounds (*x*-axis). (**g**, **h**) Summary graphs of mitotic arrest and cell death potency for all compounds. *x*-axis is drug concentration in *μ*M scale and *y*-axis represents the number of compounds within each category. Note that 56 compounds showed a mitotic arrest IC_50_ below 500 nM. (**b**–**h**) See also Supplementary Table 4

Due to the large number and chemical diversity of M-phase inhibitors, the current lack of chemical probes to study cell division and the need for novel antimitotics, we focused on the detailed characterization of mitotic inhibitors. To assess the potential of the 266 mitotic inhibitors as anti-cancer agents, they were re-synthesized and the 211 that passed quality control (Mitotic Inhibitor 1-211) were tested for their ability to arrest cells in mitosis and to decrease cancer cell viability (Figure 3a). For mitotic arrest assays, HeLa cells were treated with a 20.2-fold-titration (190pM to 10 *μ*M) of each compound for 20 h and the half maximal inhibitory concentration (IC_50_) was derived for each compound using the cell cycle profile assay used in the initial screen (Figures 3b and c and Supplementary Table 4). As an example, compound ASN05941236 displayed a mitotic arrest IC_50_ of 3.34 *μ*M (Figure 3b). Within the titration series, all compounds displayed a percent mitotic arrest between 48.3 and 83.6% (Figure 3d and Supplementary Table 4). For viability assays, cells were treated with a 14.2-fold-titration (12.2 nM to 100 *μ*M) of each compound for 72 h and cell viability was measured using the CellTiter-Glo luminescent cell viability assay (Figures 3e and f and Supplementary Table 4). As an example, compound ASN05941236 displayed a cell death IC_50_ of 3.38 *μ*M (Figure 3e). Interestingly, all 211 compounds arrested cells in mitosis and decreased cell viability (Supplementary Table 4). Most of these compounds were potent; 16 had a mitotic arrest IC_50_ of ≤100 nM, 56 had ≤500 nM, and 98 had ≤1 *μ*M (Figure 3g and Supplementary Table 4). Similarly, 13 compounds had a cell viability IC_50_ of ≤100 nM, 56 had ≤500 nM, and 95 had ≤1 *μ*M (Figure 3h and Supplementary Table 4).

### Multiparametric phenotypic analyses of M-phase inhibitors

**Figure 4:**
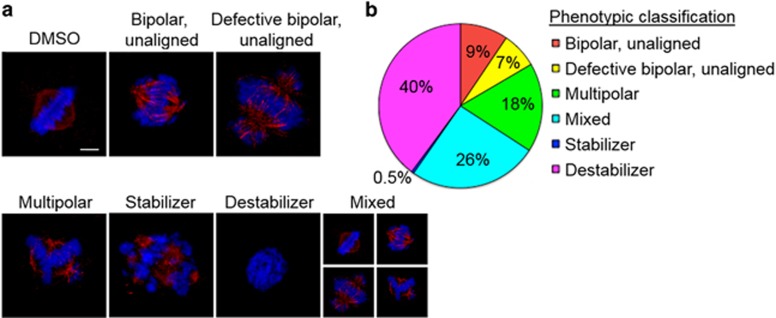
High-resolution phenotypic analysis of mitotic inhibitors. (**a**) Summary of the six phenotype classes observed upon treatment with MI-1 through MI-211: bipolar spindle with unaligned chromosomes, defective bipolar spindle with unaligned chromosomes, multipolar spindle, mixed phenotype, depolymerized microtubules, and stabilized microtubules; imaged by immunofluorescence microscopy. Cells were treated with compounds for 20 h, fixed, and co-stained for α-tubulin (anti-tubulin antibodies, red) and DNA (Hoechst 33342, blue). Bar indicates 5 *μ*m. (**b**) Summary of the percentage of the total M-phase inhibitors in each phenotypic category. See also Supplementary Table 4

To investigate the mechanism of action of each anti-mitotic compound we performed high-resolution immunofluorescence (IF) microscopy to analyze the mitotic defects induced by these compounds (Figure 4). HeLa cells were treated with each of the 211 compounds at a concentration corresponding to their mitotic arrest IC_90_ for 20 h. Cells were then fixed, permeabilized, and co-stained for DNA and *α*-tubulin. Six major classes of phenotypes were observed: multipolar spindle, normal bipolar spindle with unaligned chromosomes, defective bipolar spindle with unaligned chromosomes, mixed phenotype (containing more than one of the six phenotypes), depolymerized microtubules, and stabilized microtubules (Figure 4a and Supplementary Table 4). The compound class that induced microtubule depolymerization was the most abundant with 84 compounds falling into this category, followed by the mixed phenotype (54 compounds) and the multipolar phenotype (37 compounds) (Figure 4b and Supplementary Table 4).

### Selection of MI-181 as a lead compound

**Figure 5:**
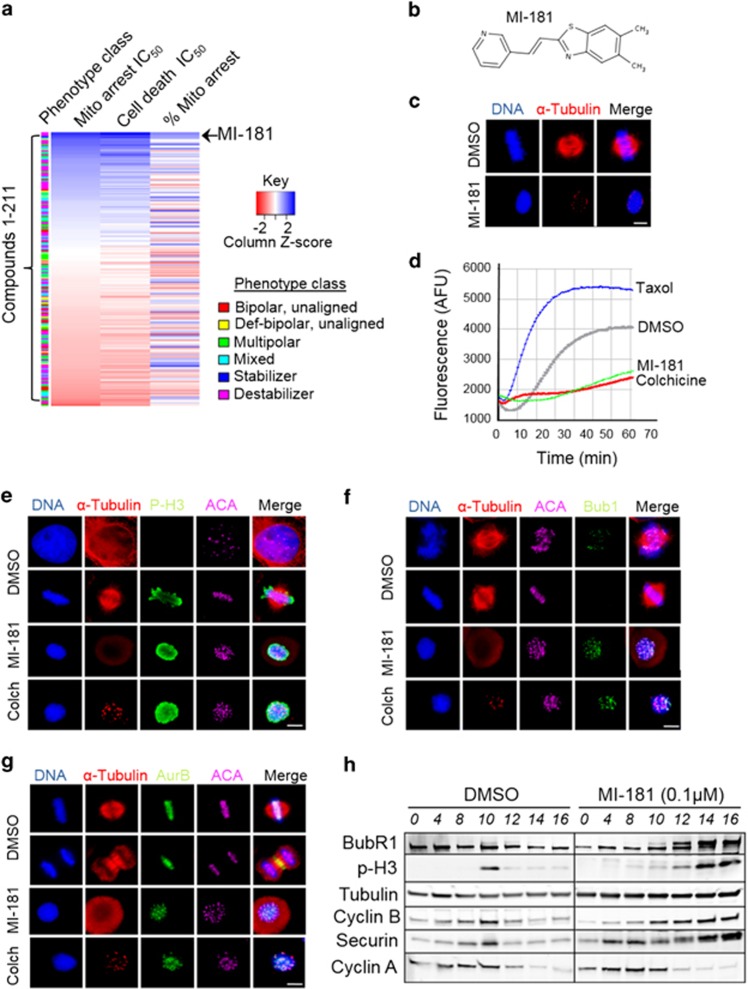
Selection of lead compound MI-181. (**a**) Clustering of 211 M-phase compounds based on their bioactive properties. For each compound the following data were grouped and the compounds were sorted based on mitotic arrest IC_50_: phenotype class, mitotic arrest IC_50_, cell death IC_50_, and percent mitotic arrest. Note that MI-181 was the most potent inhibitor (mitotic arrest IC_50_=24 nM). (**b**) Chemical structure of MI-181. (**c**) Cells were treated with DMSO or MI-181 for 20 h, fixed, co-stained for DNA (Hoechst 33342) and microtubules (anti-*α*-Tubulin antibodies), and imaged by immunofluorescence (IF) microscopy. Images show that MI-181-treated cells arrest with condensed chromosomes and depolymerized microtubules. (**d**) Results from *in vitro* tubulin polymerization reactions in the presence or absence of DMSO, MI-181, colchicine, and taxol. Note that MI-181 inhibits tubulin polymerization. (**e–g**) IF microscopy showing that MI-181-treated cells arrest in mitosis (p-H3 positive) (**e**) and activate the SAC (Bub1 remains at the kinetochore region (**f**) and AurKB remains on chromosomes and kinetochores and does not transition to the central spindle (**g**)). (**h**) Immunoblot analysis of extracts prepared from DMSO or MI-181-treated cells, which were synchronized in G1/S and released into the cell cycle. Note that cyclin A levels decrease as cells enter mitosis concomitant with an increase in p-H3 signal, and cyclin B, while BubR1 remains phosphorylated only in MI-181-treated cells, indicative of an active spindle assembly checkpoint. **(c**, **e–g**) Bar indicates 5 *μ*m

To aid the selection of lead compounds to pursue for further characterization, we performed a clustering analysis of the compounds and their bioactive properties, including mitotic arrest IC_50_, cell death IC_50_, phenotypic class of mitotic arrest, and percentage of cells arrested in mitosis (Figure 5a). This analysis revealed that MI-181 was the most potent (mitotic arrest IC_50_=23 nM and cell death IC_50_= 17 nM) compound, similar to the taxol (mitotic arrest IC_50_=37 nM and cell death IC_50_=27 nM) and colchicine (mitotic arrest IC_50_=24 nM and cell death IC_50_=12 nM) controls (Figure 5a and Supplementary Figures 1 and 2). In addition, MI-181 arrested more cells in mitosis compared with colchicine and taxol, 77% *versus* 76%, and 74%, respectively (Supplementary Table 4). MI-181 is a small (266Da) synthetic benzothiazole-based compound, and *in silico* analysis of its physiochemical properties indicated that it conformed to parameters needed for oral bioavailability in humans^16^ (Figure 5b and Supplementary Figure 3). Unsurprisingly a substructure search for FDA-approved benzothiazole-based and structurally related benzimidazole-based drugs revealed 8 benzothiazole-based drugs and 10 benzimidazole-based drugs approved for various clinical uses (Supplementary Figure 4). For example, benzothiazoles included the vasodilator Fostedil and a tetrodotoxin-sensitive sodium channel blocker Riluzole approved for the treatment of heart disease and amyotrophic lateral sclerosis, respectively (Supplementary Figure 4).^35, 36, 37, 38^ Whereas benzimidazoles included inhibitors of tubulin polymerization like nocodazole and mebendazole approved for the treatment of neoplasms and warm infestations (Supplementary Figure 4).^39,40^ Thus benzothiazoles that inhibit tubulin polymerization, like MI-181, have potential for clinical use. Although the benzothiazole and benzimidazole substructures share strong chemical similarity, the chemical diversity of FDA-approved drugs with these substructures is diverse, indicating that these substructures have been effectively used as scaffolds for generating specificity to various cellular targets for the treatment of various diseases (Supplementary Figure 4).

### MI-181 inhibits tubulin polymerization

To evaluate MI-181's mechanism of action, we treated HeLa cells with MI-181 for 20 h, fixed them, co-stained them for DNA and α-tubulin, and imaged them by IF microscopy (Figure 5c). These analyses revealed that MI-181-treated cells failed to form a mitotic spindle and only small tubulin puncta were observed, indicative of microtubule depolymerization (Figure 5c). This was further confirmed *in vitro* where MI-181 inhibited tubulin polymerization in an *in vitro* tubulin polymerization assay, similar to colchicine (Figure 5d).

### MI-181 arrests cells in mitosis, activates the SAC, and triggers cell death

To explore the nature of the MI-181-induced mitotic arrest, we asked if it was activating the spindle assembly checkpoint (SAC) to arrest cells in mitosis. First, we confirmed that MI-181 was indeed arresting cells in mitosis. HeLa cells were treated with DMSO, MI-181, or colchicine for 18 h, cells were fixed, co-stained for DNA, microtubules, kinetochores, and the mitotic marker p-H3, and imaged by fluorescence microscopy. Similar to colchicine treatment, MI-181 treatment arrested cells in mitosis with p-H3-positive staining and unaligned tightly condensed chromosomes (Figure 5e). Next, we asked if the SAC was activated in these cells by co-staining for the SAC component Bub1. Indeed, Bub1 remained localized to the kinetochore region in colchicine-and MI-181-treated cells (Figure 5f). Similarly, in cells co-stained for the SAC kinase AurKB, AurKB remained localized to the kinetochore region and never transitioned to the central spindle as in control DMSO-treated cells (Figure 5g). These data indicated that MI-181-treated cells were arrested in early mitosis with an active SAC. To further validate this, cells were synchronized in G1/S, released into the cell cycle in the presence of DMSO or MI-181 and cell extracts were prepared at several time points post release. Consistent with our fixed-cell IF results, immunoblot analysis of these extracts revealed that MI-181-treated cells arrested in mitosis (p-H3 positive), activated the SAC (BubR1 remained phosphorylated), and stabilized cyclin B while degrading cyclin A (Figure 5h). In addition, the MI-181-induced mitotic arrest was reversible, as cells exited mitosis within 2 h of drug washout (Supplementary Figure 5).

**Figure 6:**
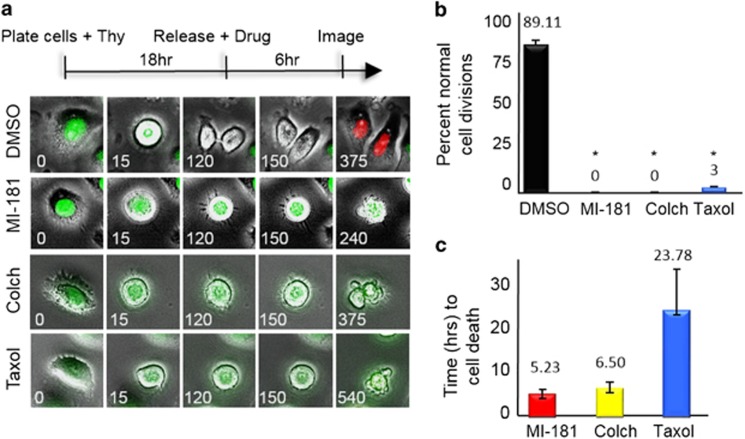
MI-181 is a potent cell division inhibitor. (**a**) Live-cell time-lapse microscopy of HeLa FUCCI cells treated with DMSO, MI-181, colchicine, or taxol. Time is in minutes. (**b**) The percentage of cells undergoing normal cell division was quantified for DMSO, MI-181, colchicine, or taxol-treated cells. Asterisk denotes *P*-value <0.0001. (**c**) The time from mitotic entry to cell death was quantified for DMSO-, MI-181-, colchicine-, or taxol-treated cells. Error bars indicate ±S.D. from the mean

To further analyze the cellular consequences of treating cancer cells with MI-181, we coupled cell synchronization with live-cell time-lapse IF microscopy. Synchronized HeLa fluorescent ubiquitination-based cell cycle (FUCCI) indicator cell line cells were treated with DMSO, MI-181, colchicine, or taxol 2 hours prior to mitotic entry and their effect on mitosis was assessed^41^ (Figure 6a). Images were captured at 15-min intervals and processed into movie format (Figure 6a and Supplementary Movies 1). The movies were then analyzed to determine the percentage of cells with a defective mitosis and the length of time between mitotic entry and cell death^42^ (Figures 6b and c). Whereas control DMSO-treated cells transitioned through mitosis (green fluorescence) and into G1 (red fluorescence) normally, MI-181-treated cells arrested in mitosis and failed to divide similar to colchicine- and taxol-treated cells (% normal cell divisions for MI-181=0, *P*<0.0001; colchicine=0, *P*<0.0001; taxol=3±0.8, *P*<0.0001; compared with DMSO=89.11±2.9) (Figure 6b). Although individual cell responses to drugs differed widely, colchicine-and MI-181-treated cells arrested for a shorter time-length than taxol prior to apoptosing (MI-181=5.3±0.96 h, colchicine=6.2±1.27 h, compared with taxol=23.78±9.03 h) (Figure 6c). These data indicated that MI-181 was a potent cell cycle-specific inhibitor, which arrested cells in mitosis, activated the SAC, and induced an apoptotic cell death with faster kinetics than taxol.

### MI-181 is active in a broad array of cancers, especially melanomas

**Figure 7:**
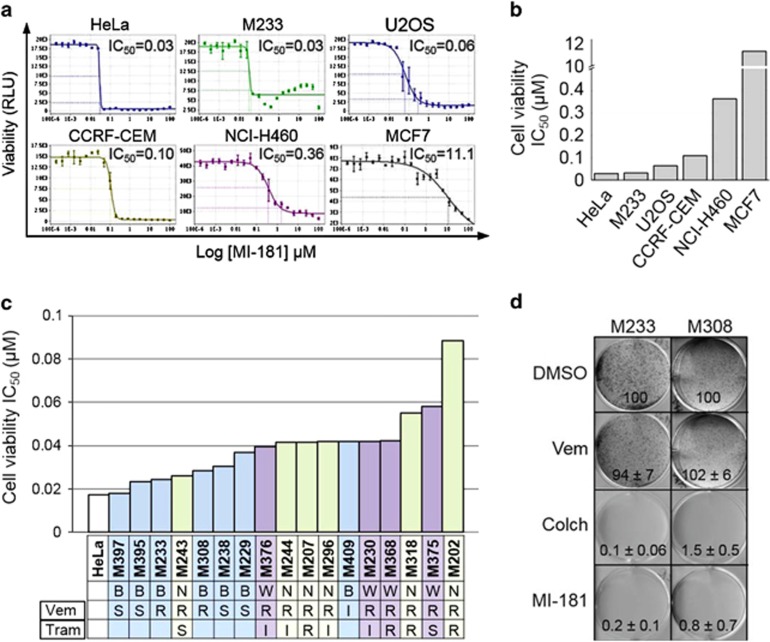
MI-181 is a potent cancer cell division inhibitor, especially melanomas. (**a**, **b**) MI-181 is potent against a broad panel of cancer cell lines. Cervical adenocarcinoma (HeLa), breast adenocarcinoma (MCF7), melanoma (M233), osteosarcoma (U2OS), acute lymphoblastic leukemia (CCRF-CEM), non-small cell lung carcinoma (NCI-H460), and breast adenocarcinoma (MCF7) cells were treated with increasing concentrations of MI-181 (190pM-10 *μ*M) for 72 h and their cell viability IC_50_ was assessed using the CellTiter-Glo assay. (**c**) A panel of melanoma cells were treated with increasing concentrations of MI-181 and the IC_50_ was determined for each cell line as described in **a**. B, BRAF*^V600E^*; N, NRAS*^Q61L^*; R, resistant; S, sensitive; Tram, Trametinib; Vem,Vemurafenib; W, wildtype. (**d**) MI-181 inhibits melanoma colony formation. M233 and M308 melanoma cells were treated with DMSO, Vemurafenib (1 *μ*M), colchicine (100 nM), or MI-181 (100 nM) for 7 days and the percent colony formation, normalized to DMSO, was quantified. Percent colony formation and standard deviation are indicated at the bottom of each panel for each drug and cell line

To determine if MI-181 had broad anti-cancer activity, we treated a diverse panel of cancer cell lines including cervical adenocarcinoma (HeLa), breast adenocarcinoma (MCF7), melanoma (M233), osteosarcoma (U2OS), acute lymphoblastic leukemia (CCRF-CEM), non-small cell lung carcinoma (NCI-H460), and breast adenocarcinoma (MCF7) with MI-181 and determined its cell viability IC_50_ (Figures 7a and b). Interestingly, MI-181 showed great efficacy across most cancer cell lines with a cell viability IC_50_ ranging from 0.03 to 0.36 *μ*M, with the exception of MCF7 cells (IC_50_=11 *μ*M) (Figures 7a and b). These results indicated that MI-181 was potent across a broad array of cancers and was most effective against cervical adenocarcinoma and melanoma cell lines. Therefore, we analyzed the efficacy of MI-181 in a panel of melanoma cell lines with defined genetic backgrounds including BRAF^*V600E*^ and NRAS^*Q61L*^ mutations and varied sensitivities to Vemurafenib (BRAF inhibitor) and Trametinib (MEK inhibitor), which are currently used to treat BRAF^*V600E*^ melanomas^43,44^ (Figure 7c). MI-181 displayed great potency across this panel (IC_50_=18–90 nM) (Figure 7c and Supplementary Table 5). As a general trend BRAF^*V600E*^ cell lines were slightly more sensitive than NRAS^*Q61L*^ cell lines and MI-181 was effective in Vemurafenib- and Trametinib-resistant cell lines (Figure 7c and Supplementary Table 5). Finally, we tested the ability of MI-181 to inhibit melanoma colony formation using the M233 and M308 cell lines (both resistant to Vemurafenib). Indeed, MI-181 was a potent inhibitor of colony formation (percentage colony formation for 10 nM MI-181=0.2±0.1 and 0.8±0.7; for 10 nM colchicine=0.1±0.06 and 1.5±0.5; and for 10 nM Vemurafenib= 94±7 and 102±5) (Figure 7d). Thus, MI-181 is a potent inhibitor of melanoma cell lines.

## Discussion

Chemical inhibition of the cell cycle and cell division has been a fruitful approach for understanding the mechanisms that cancer cells rely on to proliferate and for the development of therapeutics to inhibit these processes. Previous studies taking unbiased chemical screening approaches to identify anti-proliferative agents were limited by screening assay output, chemical library composition, lack of chemical analysis on hits, and a narrow focus on specific phases of the cell cycle. Here, we devised a cell cycle profiling high-throughput chemical screening approach that enabled the identification of cell cycle modulators specifically inhibiting G1, S, G2, and M-phases while avoiding the difficulties associated with high-throughput fluorescence activated cell sorting screening. The utility of this approach was validated by the identification of well-validated cell cycle-specific inhibitors and their analogs. Most importantly, this screen uncovered numerous novel compounds representing variable chemotypes, which warrant further validation and characterization as anti-cancer agents.

In our approach, the use of computational chemoinformatics enabled the generation of hit compound network similarity graphs that grouped compounds based on chemotypes, facilitated compound target and off-target prediction, and identified analogs of validated inhibitors with the potential to be more potent, more specific, or less toxic. The wealth of information derived from these computational analyses could aid future studies aimed at discovering/developing anti-cancer agents. Thus, we propose that similar chemical deconvolution approaches be part of every future high-throughput chemical screen. Similarly, the characterization of compounds based on potency and multiparametric phenotypic analysis adds valuable information to the selection of lead compounds for the purposes of being used as chemical probes to dissect the mechanisms driving the cell cycle, as anti-cancer agents, or both and should be considered an integral part of cell-based chemical screening campaigns.

The chemical diversity of the mitotic inhibitors and the array of mitotic defects induced by these compounds indicate that they are likely targeting a broad array of mitotic targets. Therefore, these inhibitors could be used to study the function of these targets in an acute and temporal manner and they warrant further evaluation and target identification/validation. The selection of MI-181 as a lead anti-cancer compound in our study highlights the utility of cell-based chemical screening for the identification of potent cell permeable drug-like phase specific drugs. MI-181 targets tubulin, inhibits tubulin polymerization, activates the SAC, arrests cells in mitosis, and triggers an apoptotic cell death. Most importantly, MI-181 has broad anti-cancer activity and is especially potent against melanomas.

Microtubule depolymerizers like vinblastine and vincristine are currently in clinical use for the treatment of testicular cancer, lung cancer, leukemias, and lymphomas^45^ and there is a critical need to identify novel synthetic molecules that can address the limitations of these drugs (synthesis, toxicity, resistance, and so on). Thus, MI-181 represents an opportunity to develop improved alternatives to these drugs.

## Materials and Methods

### Cell culture

Non-melanoma cell lines were purchased from ATCC (Manassas, VA, USA), which verified identity by short-tandem repeat profiling, and were passaged for <6 months following receipt and were maintained in F12 : DMEM 50  :  50 medium (GIBCO, Grand Island, NY, USA) with 10% FBS, 2 mM L-glutamine, and antibiotics, in 5% CO_2_ at 37 °C. Melanoma cell lines were established from patient biopsies under UCLA IRB approval #02-08-067, as described previously.^46^ Melanoma cell lines were genotyped using Oncomap3 platform for 33 genes, Affymetrix Gene Chip for SNP and IonTorrent for next-generation sequencing, and were passaged for <6 months following verification, and were maintained in RPMI (GIBCO) with 10% FBS and antibiotics in 5% CO_2_ at 37 °C, as described previously.^46^ For G1/S arrests, cells were treated with 2 mM thymidine (Sigma-Aldrich, St. Louis, MO, USA) for 18 h.

### High-throughput cell cycle modulator assay

HeLa cells were plated in 384-well plates (1500 cells/well) and treated with 10 *μ*M drugs for 20 h. Cells were fixed and stained with 5 *μ*M Vybrant DyeCycle Green (Invitrogen, Grand Island, NY, USA) for 1 h at room temperature and plates were scanned with an Acumen eX3 (TTP Labtech, Cambridge, MA, USA) fluorescence cytometer using its 488 nm laser and a cell cycle histogram profile was generated for each well. For the G2/M secondary screen, 20 h post drug addition cells were fixed with 4% paraformaldehyde, permeabilized with 0.2% Triton X-100/PBS, and stained with Alexa-488-phospho-histone-H3 (Ser10, Cell Signaling, Danvers, MA, USA) and 1 *μ*g/ml Hoechst 33342 for 1 h. Plates were imaged with an ImageXpress Micro (Molecular Devices, Sunnyvale, CA, USA) high-content fluorescence microscope. Data analysis was performed using the Collaborative Drug Discovery (CDD, Burlingame, CA, USA; www.collaborativedrug.com) software and outputs were exported to Excel. The quality of the screen was assessed by calculating the Z' factor (Z' factor = 1–3 x (*σ*_p_+*σ*_n_)/(|*μ*_p_−*μ*_n_|)), which takes into account the dynamic range of the assay and variance of the data.^47^ The screen performed with an average plate Z' factor of 0.51±0.09, within the optimal performance range of 0.5–1.^47^

### Compound potency

For mitotic arrest IC_50_s, cells were treated with a 20.2-fold-titration (190pM to 10 *μ*M) of each compound for 20 h. For cell viability IC_50_s, cells were treated with a 14.2-fold-titration (12.2 nM to 100 *μ*M). Mitotic arrest IC_50_ was determined by measuring the percent G2/M arrest using the Vybrant DyeCycle Green (Invitrogen) assay described above. Cell viability IC_50_ was determined using the CellTiter-Glo Assay (Promega, Madison, WI, USA), which measures total ATP levels. Plates were read with a Tecan M1000 micro-plate reader (Tecan, San Jose, CA, USA) at 540 nm. The CDD software (Burlingame, CA, USA, www.collaborativedrug.com) was used for generating IC_50_ and IC_90_ values.

### IF and time-lapse microscopy

IF microscopy was carried out as described previously.^48^ Except that images were captured with a Leica DMI6000 microscope (Leica Microsystems, Buffalo Grove, IL, USA) and deconvolved with Leica deconvolution software. Time-lapse microscopy was performed as described previously.^18^ Briefly, HeLa FUCCI (where S through M-phase cells are green due to expression of the mAG–hGeminin fusion protein, and G1-phase cells are red due to expression of the mKO2-hCdt1 fusion protein) cells were released from G1/S in the presence of indicated drug or control DMSO, and ten Z-stack images (0.9 *μ*m steps) were captured 6 h post release at 15-min intervals. Images were deconvolved and converted to AVI movie files.

### CSNAP chemical analysis

CSNAP was used to predict the targets of G1, S, and G2-phase inhibitors as described previously. Briefly, compounds were queried in the annotated ChEMBL database version 18 (The EMBL-European Bioinformatics Institute, Cambridge, UK) using the following search parameters: tanimoto cutoff=0.75, *Z*-score cutoff=2.5. The ChEMBL target annotations were retrieved from the database based on the following criteria: confidence score=4, assay-type=binding. Finally, chemical similarity networks and ligand–target interaction fingerprint analyses were analyzed using Cytoscape (Cytoscape Consortium, San Diego, CA, USA; www.cytoscape.org) and the R statistical package (R Foundation Institute for Statistics and Mathematics, Wien, Austria; http://www.www.r-project.org), respectively.

### Clonogenic assays

M233 and M308 melanoma cells were seeded at 15 000 cells/well in six-well plates. The next day, cells were treated with indicated drugs or DMSO. Seven days post incubation, cells were fixed with 4% paraformaldehyde, stained with 0.05% Crystal violet, and colonies were counted for each treatment.

### *In vitro* tubulin polymerization assays

Tubulin polymerization reactions were carried out according to the manufacturer (BK011P, Cytoskeleton, Denver, CO, USA) in the presence of 3 *μ*M colchicine, MI-181, taxol, or DMSO. Polymerization was monitored with a Tecan M1000 micro-plate reader (Tecan) at 420 nm for 70 min at 37 **°**C.

### Antibodies

Phospho-histone-H3-488 (Cell Signaling), Phospho-histone-H3 (p-H3, Millipore, Billerica, MA, USA) *α*-tubulin (Serotec, Raleigh, NC, USA), AurKB (BD Transduction, San Jose, CA, USA), Anti-Centromere-Antibodies (ACA, Cortex Biochem, Madison, WI, USA), cyclin A and B (Santa Cruz Biotechnology, Dallas, TX, USA), and SECURIN (Gene Tex, Irvine, CA, USA). BubR1 and Bub1 were from Hongtao Yu. FITC-, Cy3-, and Cy5-conjugated secondary antibodies were from Jackson Immuno Research.

## Abbreviations

p-H3: phospho-histone H3
CSNAP: Chemical Similarity Network Analysis Pulldown
SAC: spindle assembly checkpoint
IC_50_: half maximal inhibitory concentration
