# Supplementary material for: Chemical dissection of the cell cycle: probes for cell biology and anti-cancer drug development
Source: Cell Death Dis. 2014 Oct 16;5(10):e1462–. doi: 10.1038/cddis.2014.420 (PMC4237247; doi:10.1038/cddis.2014.420)
Supplement: Supplementary Material [file cddis2014420x1.doc]

**supplementary Information**

**Chemical dissection of the cell cycle: probes for cell biology and anticancer drug development**

Silvia Senese^1^, Yu-Chen Lo^1,2^, Dian Huang^2^, Thomas A. Zangle^2^, Ankur A. Gholkar^1^, Lidia Robert^3^, Blanca Homet^3^, Antoni Ribas^3,4,5,6^, Matthew K. Summers^7^, Michael A. Teitell^2,6,8,9,10,11^, Robert Damoiseaux^10^ and Jorge Z. Torres^1,6,11*^

**SUPPLEMENTARY FIGURES**

**Supplementary Figure 1.** MI-181 chemical information.

**Supplementary Figure 2.** HeLa cell mitotic arrest and cell viability dose response curves for nocodazole, colchicine, taxol and MI-181.

**Supplementary Figure 3.** *In silico* prediction of ADMET properties for colchicine, taxol and MI-181.

**Supplementary Figure 4.** Substructure search for FDA approved benzothiazole-based and structurally related benzimidazole-based drugs.

**Supplementary Figure 5.** MI-181 is a reversible mitotic inhibitor.

**SUPPLEMENTARY TABLES**

**Supplementary Table 1.** Small-molecule high-throughput screening data.

**Supplementary Table 2.** High-throughput cell cycle profiling data.

**Supplementary Table 3.** Chemical similarity network analysis pulldown (CSNAP).

**Supplementary Table 4.** Potency and phenotypic data for antimitotic compounds.

**Supplementary Table 5.** MI-181 melanoma cell line screening data.

**SUPPLEMENTARY MOVIES**

**Supplementary Movie 1.** DMSO-treated control cell undergoing mitosis.

**Supplementary Movie 2.** MI-181-treated cell undergoing mitosis.

**Supplementary Movie 3.** Colchicine-treated cell undergoing mitosis.

**Supplementary Movie 4.** Taxol-treated cell undergoing mitosis.

**SUPPLEMENTARY FIGURES**

**
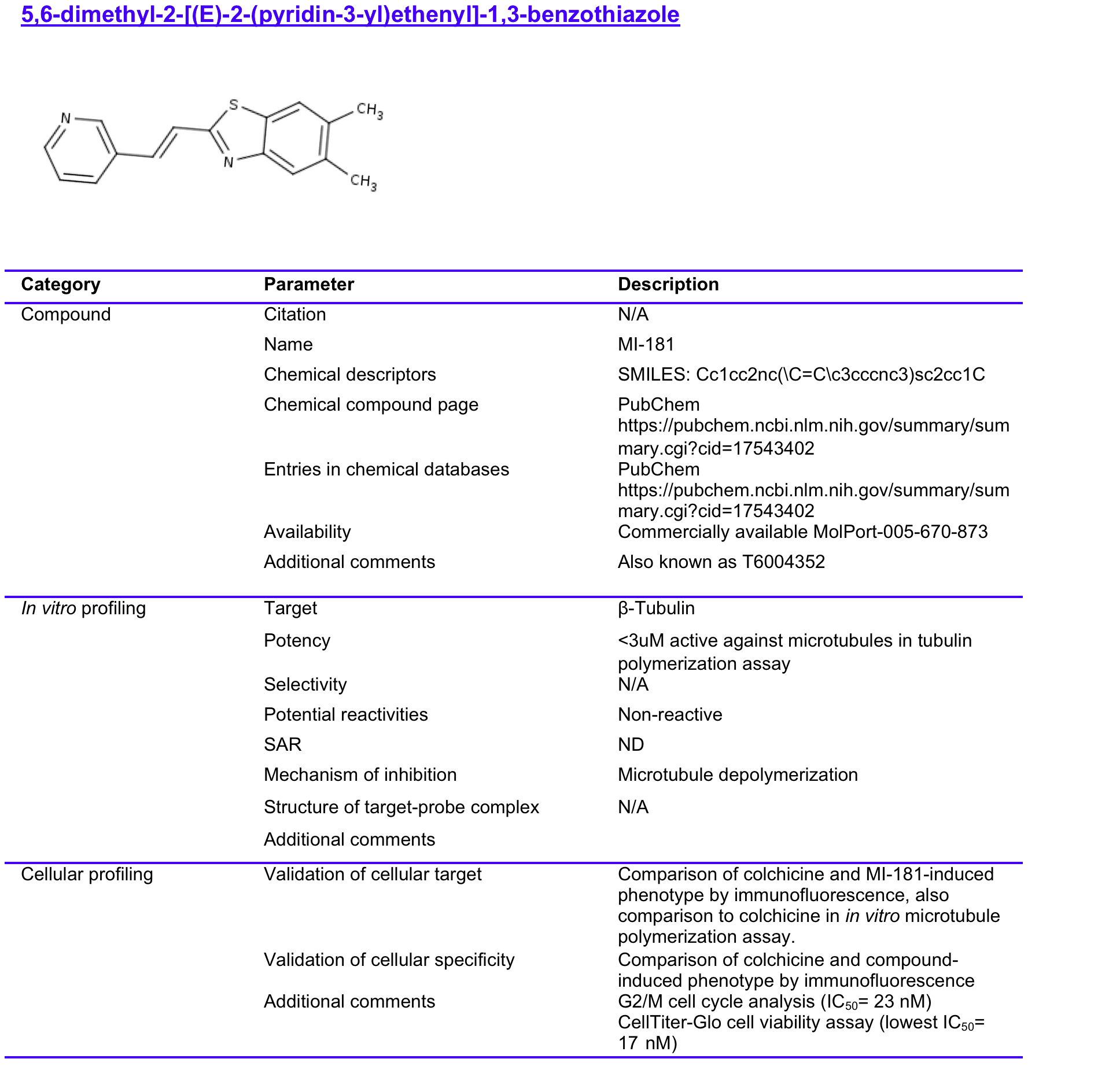
**

**Supplementary Figure 1.** MI-181 chemical information.

**Supplementary Figure 2.** HeLa cell mitotic arrest and cell viability dose response curves for nocodazole, colchicine, taxol and MI-181.

**Supplementary Figure 3.** *In silico* prediction of ADMET properties for colchicine, taxol and MI-181.

**
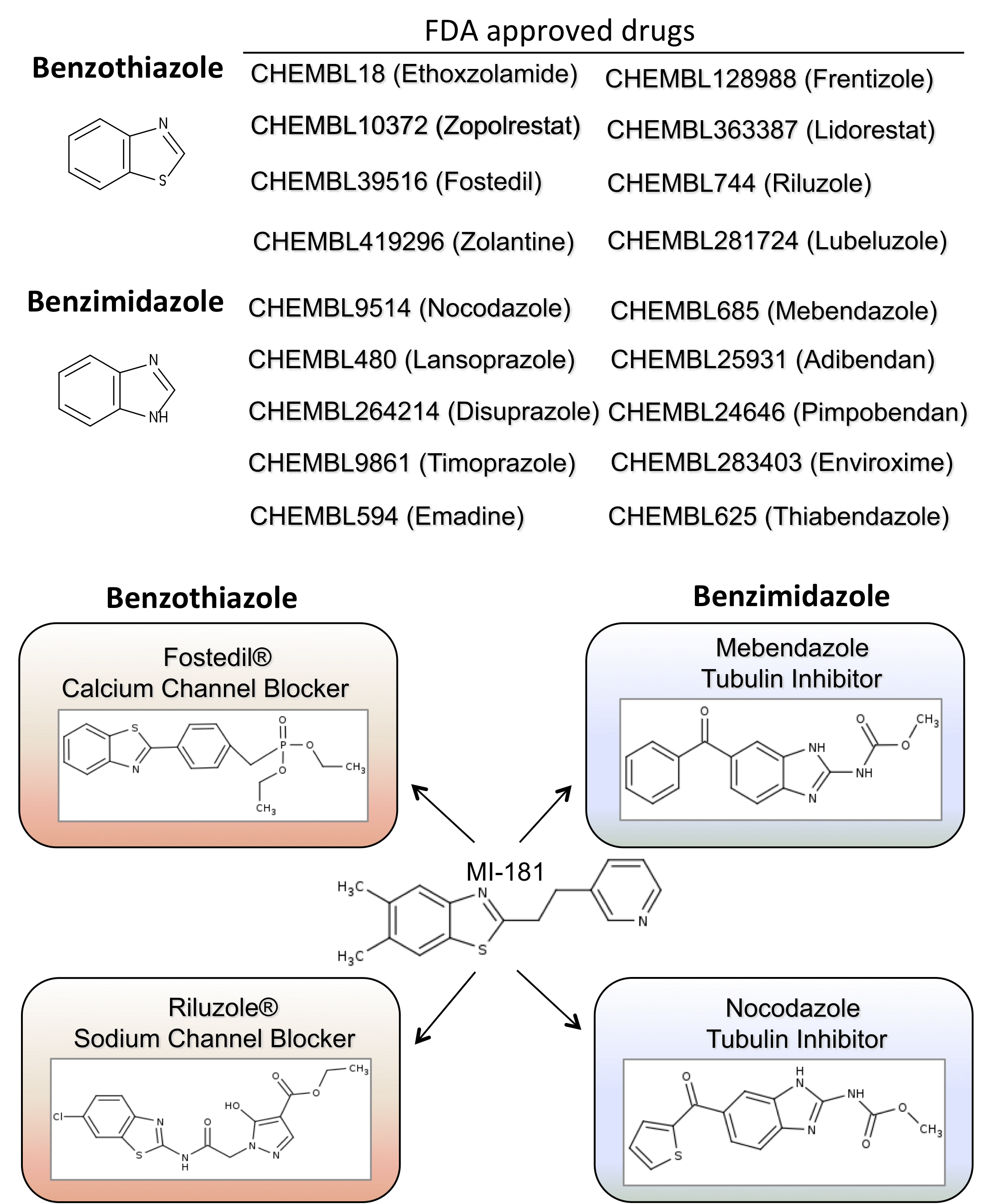
**

**Supplementary Figure 4.** Substructure search for FDA approved benzothiazole-based and structurally related benzimidazole-based drugs.

**
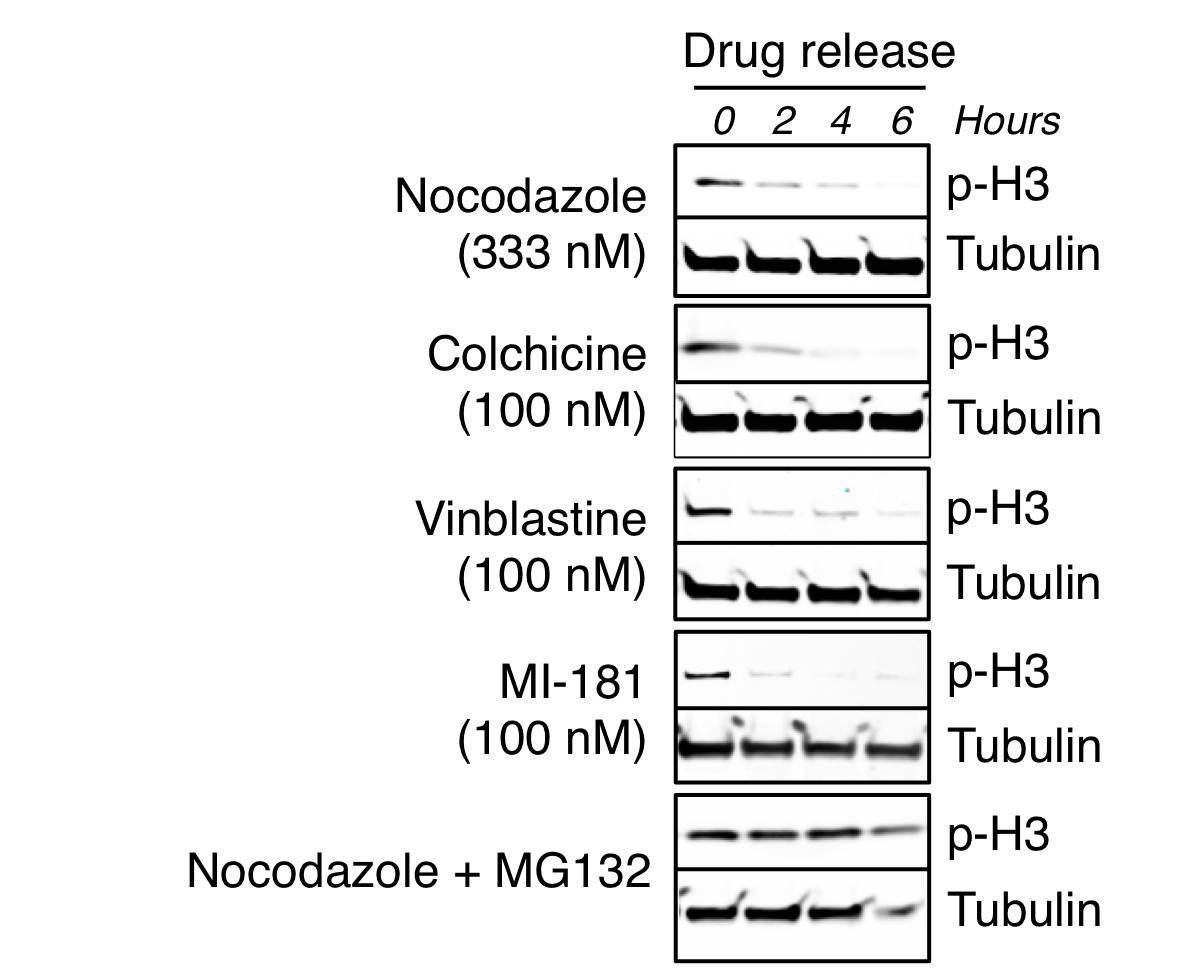
**

**Supplementary Figure 5.** MI-181 is a reversible mitotic inhibitor. HeLa cells were treated with the indicated drugs for 18 hours. Cells were then washed and released into the cell cycle. Protein samples were prepared at the indicated time points and were analyzed by immunoblotting with anit-p-H3 and anti-α-Tubulin antibodies.

**SUPPLEMENTARY TABLES**

| **Category** | **Parameter** | **Description** |
| --- | --- | --- |
| Assay | Type of assay | Cell-based |
|  | Target | Cell cycle modulators |
|  | Primary measurement | Cell cycle profile, detection of G1, S, G2, and G2/M arrest |
|  | Key reagents | Vybrant DyeCycle Green Stain (Invitrogen)  p-H3-488 Antibody (Cell Signaling) |
|  | Assay protocol | See Online Methods |
|  | Additional comments |  |
| Library | Library size | 79,827 compounds |
|  | Library composition | Drug-like molecules |
|  | Source | UCLA Molecular Screening Shared Resource |
|  | Additional comments |  |
| Screen | Format | 384-well plates |
|  | Concentration(s) tested | 10μM, <1% DMSO |
|  | Plate controls | Internal controls DMSO, Taxol |
|  | Reagent/ compound dispensing system | **Biomek FX (Beckman Coulter)** and Multidrop **384** (Thermo LabSystems) liquid handlers |
|  | Detection instrument and software | Acumen eX3 (TTP Labtech) |
|  | Assay validation/QC | Z’ score 0.51 ± 0.09 |
|  | Correction factors |  |
|  | Normalization | To internal controls DMSO and Taxol |
|  | Additional comments |  |
| Post-HTS analysis | Hit criteria | G1-phase inhibitors (>4 STDs from the mean), S-phase inhibitors (> 5 STDs from the mean), G2/M inhibitors (>67% G2/M arrest) |
|  | Hit rate | Total 0.613% cell cycle modulator hit rate = 0.613%; 0.086% G1-phase inhibitors, 0.185% S-phase inhibitors, 0.009% G2-phase inhibitors and 0.333% M-phase inhibitors |
|  | Additional assay(s) | G2/M deconvolution screen for p-H3-488 antibody positives cells, CellTiter-Glo luminescent cell viability assay and immunofluorescence microscopy-based multiparametric phenotypic analyses. |
|  | Confirmation of hit purity and structure | Compounds were repurchased from MolPort and compound structure and purity were verified analytically |
|  | Additional comments |  |

**Supplementary Table 1.** Small-molecule high-throughput screening data. Summary of screening assays, chemical library, screening conditions and post-HTS analysis.

**Supplementary Table 2.** High-throughput cell cycle profiling data.

Excel file with G1, S, G2 and M-phase inhibitor compound name and percent arrest. Standard deviations from the mean for each phase are indicted.

**Supplementary Table 3.** Chemical similarity network analysis pulldown (CSNAP).

Excel file with CSNAP analysis of the top G1, S, and G2-phase inhibitors.

**Supplementary Table 4.** Potency and phenotypic data for antimitotic compounds.

Excel file listing M-phase inhibitors by compound name, mitotic arrest IC_50_, cell viability IC_50_, and mitotic phenotype classification.

**Supplementary Table 5.** MI-181 melanoma cell line screening data.

Excel file listing cell viability IC_50_ for MI-181 across a panel of melanoma cell lines.

**SUPPLEMENTARY MOVIES**

**Supplementary Movie 1.** DMSO-treated control cell undergoing mitosis.

Live cell time-lapse microscopy of control DMSO-treated cells. HeLa-FUCCI cells were arrested with Thymidine for 18 hours, washed, released into fresh media and DMSO was added 6 hours post release. Images from 3 channels (phase contrast, FITC and Cy3) were captured every 15 minutes at 20X magnification with a Leica DMI6000 microscope and processed using Leica deconvolution software (Leica Microsystems) and converted to an AVI movie. Each frame represents a fifteen-minute interval.

**Supplementary Movie 2.** MI-181-treated cell undergoing mitosis.

HeLa-FUCCI cells were synchronized, treated with MI-181, and imaged by live time-lapse microscopy as described for Supplementary Movie 1.

**Supplementary Movie 3.** Colchicine-treated cell undergoing mitosis.

HeLa-FUCCI cells were synchronized, treated with colchicine, and imaged by live time-lapse microscopy as described for Supplementary Movie 1.

**Supplementary Movie 4.** Taxol-treated cell undergoing mitosis.

HeLa-FUCCI cells were synchronized, treated with taxol, and imaged by live time-lapse microscopy as described for Supplementary Movie 1.
